# Case-based surveillance of antimicrobial resistance with full susceptibility profiles

**DOI:** 10.1093/jacamr/dlz070

**Published:** 2019-12-10

**Authors:** Sukhyun Ryu, Benjamin J Cowling, Peng Wu, Scott Olesen, Christophe Fraser, Daphne S Sun, Marc Lipsitch, Yonatan H Grad

**Affiliations:** 1 WHO Collaborating Centre for Infectious Disease Epidemiology and Control, School of Public Health, Li Ka Shing Faculty of Medicine, The University of Hong Kong, Hong Kong Special Administrative Region, China; 2 Department of Preventive Medicine, College of Medicine, Konyang University, Daejeon, Republic of Korea; 3 Department of Immunology and Infectious Diseases, Harvard T.H. Chan School of Public Health, Boston, MA, USA; 4 Big Data Institute, Nuffield Department of Medicine, University of Oxford, Oxford, UK; 5 Center for Communicable Disease Dynamics, Department of Epidemiology, Harvard T.H. Chan School of Public Health, Boston, MA, USA; 6 Division of Infectious Diseases, Department of Medicine, Brigham and Women’s Hospital, Harvard Medical School, Boston, MA, USA

## Abstract

Surveillance of antimicrobial resistance (AMR) is essential for clinical decision-making and for public health authorities to monitor patterns in resistance and evaluate the effectiveness of interventions and control measures. Existing AMR surveillance is typically based on reports from hospital laboratories and public health laboratories, comprising reports of pathogen frequencies and resistance frequencies among each species detected. Here we propose an improved framework for AMR surveillance, in which the unit of surveillance is patients with specific conditions, rather than biological samples of a particular type. In this ‘case-based’ surveillance, denominators as well as numerators will be clearly defined with clinical relevance and more comparable at the local, national and international level. In locations with sufficient resources, individual-based data on patient characteristics and full antibiotic susceptibility profiles would provide high-quality evidence for monitoring resistant pathogens of clinical importance, clinical treatment of infections and public health responses to outbreaks of infections with resistant bacteria.

## Introduction

Antimicrobial resistance (AMR) poses a fundamental threat to global health.^1^ Growing levels of AMR among bacteria have raised the concern that some infectious diseases might become untreatable in a post-antibiotic era.^2–4^ Standardized surveillance data for AMR are necessary to understand the extent of the problem and temporal trends in resistance, in order to prioritize threats and target countermeasures. Unfortunately, such data remain unavailable in many parts of the world; the absence of standard reporting protocols undermines geographic and temporal comparisons.^5^ Here we consider the purposes of AMR surveillance and propose two considerations to enhance the value of surveillance data without adding substantially to the burden of data collection.

## Purposes of AMR surveillance

There are three major reasons for AMR surveillance. The first is to guide clinical decision-making.^6^^,^^7^ Clinical decisions about which antibiotic to use to treat a patient with bacterial infection have a number of considerations. Some are specific to the patient, including disease severity and prognosis. The risk of resistance is also an important consideration. Ideally, laboratory tests can identify the pathogen and its antibiotic susceptibility profile. In practice, treatment decisions must typically be made before the causative organism and its antibiotic susceptibility are known. AMR surveillance data, including local patterns in the frequencies of pathogens causing this disease, and the level of resistance of those pathogens to antibiotics, guide prescribers’ choice of antibiotic in these situations. Following this initial ‘empiric’ choice of treatment, antibiotic susceptibility testing is often requested by clinicians to guide treatment decisions when bacterial specimens are available and the clinical scenario warrants efforts towards tailored therapy (such as bacteraemia or recurrent urinary tract infection that may be caused by MDR organisms). Results of microbiological tests on specimens collected from the patient, including pathogen detection frequencies and antimicrobial susceptibility, can be compiled by microbiology laboratories and provided to health authorities for surveillance purposes. Clinical guidelines are often based on the information generated from these processes, i.e. laboratory testing results of diagnostic specimens, on an *ad hoc* basis rather than from any routine or systematic surveillance.

Second, AMR surveillance is useful for public health practice, because it characterizes the trends of resistant infections. Such surveillance data can be used to profile geographic patterns and temporal trends in AMR-related infections in specific settings, to guide enquiries into the factors shaping trends in resistance and to predict the potential impact of specific interventions. For this objective, it is valuable to collect information on the role of different pathogens in causing clinical syndromes and on the frequency with which each of these pathogens has a particular resistance profile: that is, its pattern of resistance and susceptibility to several drugs that could be used for treatment. Surveillance that quantifies and compares the amount of disease attributable to different resistant pathogens in different settings and locations can galvanize action to reduce this burden.

Third, AMR surveillance provides epidemiological data to study the health impact of AMR and the effectiveness of control measures in healthcare facilities and the community. Infections with drug-resistant bacteria are associated with longer hospital stays and higher mortality in patients,^8^ and antibiotic stewardship and infection control measures in hospital can reduce AMR by limiting antibiotic use.^9–11^ However, it remains challenging to assess the effectiveness of interventions on AMR because of the lack of consensus on how to define AMR-associated disease burden.

A network of AMR surveillance systems that fulfil these three main purposes should also be able to provide data for comparative analyses, drive local, national and regional strategy for AMR control and provide an evidence base for AMR action plans and advocacy.

## Sample-based surveillance as a current approach for AMR surveillance

In 2014, the WHO introduced the Global Antimicrobial Resistance Surveillance System (GLASS) to provide a more standardized approach to global AMR surveillance on a priority list of bacteria, diseases and types of specimen.^6^^,^^12^ GLASS provides guidance to standardize AMR data, which can be compared among countries and indicate global patterns and trends in resistance. GLASS collects AMR data mainly in the format of sample-based surveillance, which is based on patient specimens including blood, urine, stool, urethral samples and cervical swabs from designated laboratories for clinical purposes.^12^ An advantage of the sample-based approach over pathogen-based surveillance is the clarity in denominators, which include samples collected from target patients testing both positive and negative for pathogens of interest.^6^ However, sample-based surveillance may inhibit interpretation of resistance data because of the absence of information about possible variations in case-mix among patients from different hospitals or departments, and in clinical decisions on collecting specimens for microbiological analysis, which would both affect the characteristics of samples submitted to the surveillance laboratories.

## Case-based surveillance as a comprehensive approach to AMR surveillance

Here, we make a case for a broader and more comprehensive approach to the surveillance of antibiotic resistance, termed ‘case-based surveillance’, in contrast to existing surveillance that is often pathogen-based or sample-based.^6^^,^^12^ The basic concept of case-based surveillance is prospective surveillance of a defined population or patient group for the incidence or prevalence of infections by particular pathogens and the prevalence of resistance among identified pathogens. The denominator for case-based surveillance will be a defined population, such as people in the general community, patients having received specific clinical procedures or patients with specific conditions or characteristics. In its simplest form, case-based surveillance could focus on systematic collection of a minimum dataset on a few priority conditions such as urinary tract infection, septicaemia, etc., in contrast to current approaches, which focus on sample types such as urine, blood, etc., without corresponding clinical information or clear patient denominators. In locations with more resources, case-based surveillance could capture individual data on symptoms, laboratory tests, patient demographics and history, and provide a comprehensive picture of patterns in resistance by patient characteristics.

For community-acquired infections such as community-acquired pneumonia and sexually transmitted diseases, we envisage a population-based strategy where the occurrence of each syndrome is monitored in selected locations, relevant specimens are systematically collected and tested for pathogens and antibiotic resistance and a basic set of demographic and clinical information is collected on each patient. If resources permit, the information collected should include recent medical history including comorbidities and treatment history of antibiotics, which are associated with AMR profiles.^13^^,^^14^

For nosocomial infections such as central line-associated bloodstream infections and catheter-associated urinary tract infections, we envisage surveillance among inpatients where the denominator is the number of patient admissions with a catheter for >48 h in hospital and the numerator is the number of those patient admissions with a laboratory-confirmed bloodstream infection or urinary tract infection with pathogens of interest. Information collected for surveillance would include the potential causative bacteria identified from blood or urine culture and resistance profile of the pathogens, along with patients’ basic demographics and recent medical history including antibiotic use. This approach to surveillance of nosocomial infections is already in use in some hospitals for internal quality assurance, but does not tend to be published or shared.^15^ What we are proposing here is a continuation of such existing approaches, but with common protocols and case definitions to facilitate multidimensional comparisons.

While case-based surveillance would require additional resources compared with sample-based surveillance because of the need for curated clinical information in addition to the laboratory results, it has a number of major advantages, which we believe justify the additional resources required. First, case-based surveillance could directly link the characterized AMR profile with patients at risk of AMR infection or severe outcome of infection in different clinical settings. By collecting in a systematic prospective manner, the data obtained through case-based surveillance would allow clearer insights into AMR patterns in patients with different types of infections. Second, the data obtained by this approach would better inform treatment guidelines and clinical practice because information would be available from a systematic sample of patients with each condition of interest.^16^ Furthermore, standardized surveillance of nosocomial infections can also provide valuable information to initiate and assess interventions for hospital infection control.^17^ Third, case-based surveillance would help to identify high-risk populations and settings vulnerable to AMR infections and therefore to determine specific public health measures. Fourth, it would provide consistent and systematic data streams for analyses of the effectiveness of interventions implemented in hospitals or community settings, or at a regional or national level. Finally, by having the specific demographics of the case population as well as antibiotic prescription patterns, the linkage among the AMR phenotypes and AMR-associated disease burden could be studied. To minimize the additional logistical burden of case-based surveillance,^18^ only a subset of cases could be sampled, for example on certain days of the week in selected sentinel hospitals and clinics.^19^ The availability of electronic medical records would aid feasibility.

## Reporting full susceptibility profiles

Alongside the move toward case-based surveillance, a second change in the approach to reporting AMR data would further enhance its value: reporting full antibiotic susceptibility profiles instead of reporting susceptibility to each of the antibiotics of interest separately. Under the most common existing system for reporting susceptibility results, a surveillance system might report that 10% of *Escherichia coli* isolates are resistant to gentamicin, 18% are resistant to ciprofloxacin and 11% are resistant to third-generation cephalosporins. Naively, one might expect that the percentage resistant to all three classes would be around 0.2%, the product of these three proportions, if *E. coli* develops resistance to each of the antibiotics independently. In fact, in England, the proportion resistant to all three was around 5% during the period 2012–16, from which these numbers are derived, a fact that can be appreciated only because the English Surveillance Programme for Antimicrobial Utilisation and Resistance (ESPAUR) system, unlike many systems currently in use today, reports frequencies of specific MDR patterns, along with frequencies of resistance to individual drugs.^20^

To make such reporting routine, the ideal approach would consider all drugs tested and the susceptibility patterns responsible for more than some defined threshold proportion of cases; for five specific drugs, such patterns might be ‘RRRSS’ or ‘RSSSR’. For some drugs an intermediate level of antibiotic susceptibility may need to be reported given its clinical relevance and significance.

Full antibiotic susceptibility profiles are included in some reports^21^ and some surveillance systems, such as ESPAUR^20^ and the US CDC Gonococcal Isolate Surveillance Project^22^ but this approach is not adopted widely and routinely. Nevertheless, any system that tests isolates against a standard panel of drugs will have individual-level information on susceptibility patterns. In general, no additional testing would be required to implement full susceptibility reporting. As with a move to case-based surveillance, a move to reporting susceptibility profiles would serve many objectives of patient-centred surveillance supported by expert consensus, in particular by enhancing the value of surveillance to inform antimicrobial choice.^23^

**Figure 1.**
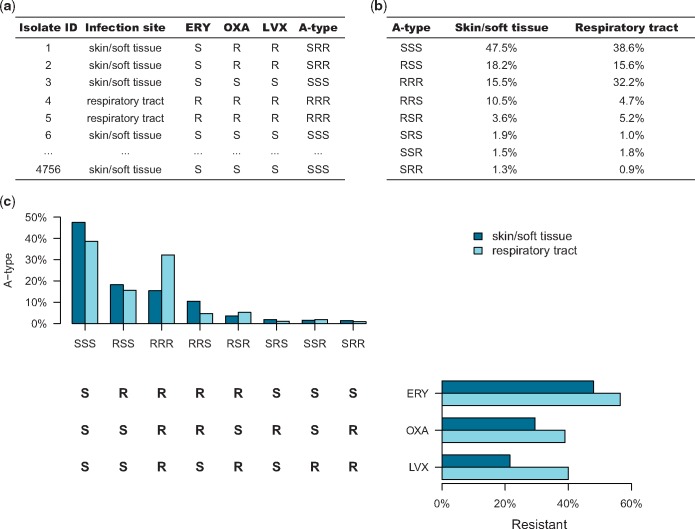
An example of case-based surveillance with antibiotic susceptibility profile of skin and soft tissue infection and respiratory tract infection. (a) Table of antibiotic susceptibility test results for a set of clinical *Staphylococcus aureus* isolates from skin and soft tissue and respiratory tract specimens. (b) The percentages of specimens from each site with each antibiogram type. (c) Histograms relating the antibiotic resistance to individual antibiotics by site and the A-types by site. ERY, erythromycin; OXA, oxacillin; LVX, levofloxacin; A-type, antibiogram type; S, sensitive; R, resistant.

Susceptibility pattern reporting also more naturally serves public health goals. Resistance to multiple drugs is a greater public health and clinical challenge than resistance to each drug individually; tracking full susceptibility patterns directly reports the frequency of MDR bacteria (Figure 1). In mathematical modelling and statistical studies of AMR, the full susceptibility pattern is the natural unit of analysis for tracking the relationship between antimicrobial use and resistance,^24^ as it allows tracking of cross-selection of resistance to one drug by use of another drug when multiple resistance is common.^25^^,^^26^ Moreover, understanding the associations between resistance to different antibiotics can enhance our understanding of the selective pressures driving resistance.^27^^,^^28^ Finally, a shift to a complete reporting of susceptibility profiles would improve our understanding of the burden and impact of AMR as a whole.

## Conclusions

AMR surveillance is critical to inform implementation and monitoring of control interventions. Sample-based surveillance strategy has provided rough estimates of the scale of AMR issues at regional and global levels. However, many shortcomings of current approaches to AMR surveillance are becoming clearer (Table 1). We believe that advancing AMR surveillance by moving to a case-based approach utilizing disease-centred clinical information with full antibiotic susceptibility profiles would substantially improve the evidence base on AMR and should be a priority.

**Table 1.** Overview of the rationale for moving to case-based surveillance of AMR with full susceptibility profiles

Perspectives	Gaps in current AMR surveillance	Potential value added	
		case-based surveillance	full susceptibility reporting
Clinical decision-making	Lack of direct link between aggregate microbiological data and specific clinical syndromes Limited information from aggregate data on isolates/samples and MDR to guide empirical treatment for individual patients	Improve knowledge of resistance profiles in patients with particular characteristics and syndromes Enhance surveillance data as evidence for tailored clinical guidelines	Provide direct estimates of the MDR patterns by type of infection Improve evidence for antibiotic selection in clinical practice
Public health practice	Insufficient information to interpret secular trends in AMR derived from isolate/sample-based surveillance data Inappropriate to use isolate/sample-based surveillance data to assess effectiveness of public health interventions because of potential biases Limited information on MDR	Provide more reliable information on AMR patterns and help to identify risk groups for resistant infections Provide more reliable information for evaluating the effectiveness of public health interventions against AMR	Facilitate microbial source-tracking of MDR bacteria Improve reporting for the incidence of AMR-related diseases by patient characteristic
Epidemiological research	Lack of critical information for further use of isolate/sample-based surveillance data in epidemiological analysis Incomparability of AMR patterns identified within and across settings because of different sampling, testing and reporting practices Potentially misleading public health interpretations of data	Facilitate epidemiological studies of risk factors for development of resistance and modelling studies of resistance dynamics	Provide clearly defined numerators and denominators for tracking AMR dynamics Facilitate better understanding of the association between resistance profiles and consumption of individual antibiotics or groups of antibiotics

## Supplementary Material

dlz070_Supplementary_Data
